# Protection of a Single Dose West Nile Virus Recombinant Subviral Particle Vaccine against Lineage 1 or 2 Strains and Analysis of the Cross-Reactivity with Usutu Virus

**DOI:** 10.1371/journal.pone.0108056

**Published:** 2014-09-17

**Authors:** Teresa Merino-Ramos, Ana-Belén Blázquez, Estela Escribano-Romero, Rodrigo Cañas-Arranz, Francisco Sobrino, Juan-Carlos Saiz, Miguel A. Martín-Acebes

**Affiliations:** 1 Departamento de Biotecnología, Instituto Nacional de Investigación y Tecnología Agraria y Alimentaria (INIA), Madrid, Spain; 2 Departamento de Virología y Microbiología, Centro de Biología Molecular Severo Ochoa (CSIC-UAM), Cantoblanco, Madrid, Spain; University of Texas Medical Branch, United States of America

## Abstract

West Nile virus (WNV) is a neurovirulent mosquito-borne flavivirus. High WNV virulence was mainly associated with lineage 1 strains, but recent outbreaks have unveiled circulation of highly virulent lineage 2 strains. Co-expression of flavivirus prM and E glycoproteins drives the assembly of recombinant subviral particles (RSPs) that share antigenic features with virions. Mouse immunization with lineage 1 WNV RSPs induced a potent humoral response against WNV with production of neutralizing antibodies. A single inoculation of RSPs formulated with Al(OH)_3_ as adjuvant protected mice against a lethal challenge with WNV strains from lineage 1 or 2. The cross-reactivity of the response elicited by these RSPs was analyzed against the related flavivirus Usutu virus (USUV), which shares multiple ecological and antigenic features with WNV. Immunization with WNV-RSPs increased specific, although low, antibody titers found upon subsequent USUV infection.

## Introduction

The genus *Flavivirus* within the *Flaviviridae* family comprises more than 50 species of positive strand RNA viruses (http://www.ictvonline.org/virusTaxonomy.asp). Flaviviruses constitute a group of arboviruses including important human and animal pathogens as Yellow fever virus (YFV), Dengue virus (DENV), Tick-borne encephalitis virus (TBEV), Japanese Encephalitis virus (JEV) or West Nile virus (WNV), as well as other neglected pathogens of currently increasing interest, such as Usutu virus (USUV) [1], [2]. Both WNV and USUV are mosquito-borne flaviviruses that share in nature an enzootic infectious cycle between avian hosts and ornithophilic mosquito vectors [3], [4]. Due to different factors, including globalization of travel and trade, changes in land use, climate warming and changes in vector ecology, these flaviviruses have emerged in areas where they were not previously detected originating outbreaks in humans, horses or birds [1], [5], [6], [7], [8]. Currently, there is no vaccine or specific therapy licensed in humans for either WNV or USUV, although different WNV vaccine candidates have been approved for veterinary use and multiple innovative approaches are being developed [9], [10], [11].

WNV infects a wide range of vertebrate hosts. Although most infections in humans are asymptomatic, WNV can induce a variety of clinical signs that range from a mild flu-like febrile illness (West Nile fever) to a severe neuroinvasive disease that can be fatal [12], [13]. In fact, a high proportion of patients that recover from neuroinvasive disease carry severe long lasting sequelae [13]. Remarkably, the size and severity of WNV outbreaks has increased in the last years, in some cases associated with viral strains from lineage 2 WNV either in Africa, where this viral lineage was endemic, or in Europe, where it had not been previously detected [14], [15], [16], [17], [18]. Regarding USUV infection, the case record is rather limited since it has been only circulating in Africa until 2001, when it emerged in Europe [4], [8]. This fact together with that symptoms induced by USUV greatly vary (including fever, rash, jaundice or meningoencephalitis) presumably may have impaired its diagnosis in poorly industrialized countries, where its circulation could be underestimated [4]. Indeed, almost no attention was paid to this virus until it emerged in Europe causing avian mortality. However, since then, there is increasing evidence of USUV circulation not only among mosquitoes and birds, but also among horses [19], [20], [21] and humans [22], [23], [24], including the report of severe neuroinvasive infections [25], [26], [27]. These findings along with the similarities between USUV and WNV ecology emphasize the need to be cautious about the potential of USUV, and other emerging flaviviruses, as a threat to human or animal health [1], [2], [4], [28].

Flavivirus virions are spherical particles about 50 nm in diameter that contain a viral core constituted by the viral RNA associated to the capsid protein C enclosed into a lipid envelope that contains the two structural glycoproteins of the virion: M (cleaved premembrane, prM) and E (envelope) [29]. The E glycoprotein displays the major antigenic determinants of the virion, being the target for most neutralizing antibodies. Both WNV and USUV share common antigenic features between them and with other flaviviruses included in the JEV serocomplex, which is reflected in the potential to induce cross-reactive antibodies [29], [30]. The antibody cross-reactivity between different flaviviruses is disadvantageous for diagnostic purposes because it hampers identification of a specific pathogen through serologic tests [3], [13]. Nevertheless, cross-reactivity can be advantageous to expand the protection spectrum of flavivirus vaccines against different but antigenically related pathogens within the JEV serocomplex [31]. However, to our knowledge no previous studies addressing the cross-reactivity with USUV of the humoral response induced by any WNV-vaccines have been reported.

Co-expression of the flavivirus prM and E glycoproteins induces formation of virus-like particles commonly referred to as recombinant subviral particles (RSPs) that, despite their reduced diameter (around 30 nm), share multiple common antigenic and immunogenic properties with whole virions [32], [33], [34], [35]. Indeed, initial approaches to test the potential use of RSPs as vaccine candidates have been assessed with TBEV [36], JEV [37], Murray Valley encephalitis virus (MVEV) [38] or WNV [33]. In this study we have developed a cell line stably transfected with a plasmid expressing the WNV prM and E proteins that constitutively secreted WNV-RSPs to the culture medium. Immunization with a single dose of these RSPs formulated with Al(OH)_3_ as adjuvant protected mice against lethal challenge with lineage 1 WNV, lineage 2 WNV and promoted a cross-reactive humoral response against USUV.

## Materials and Methods

### Ethics statement

All animals were handled in strict accordance with the guidelines of the European Community 86/609/CEE at the biosafety animal facilities of the Instituto Nacional de Investigación Agraria y Alimentaria (INIA). The protocols were approved by the Committee on Ethics of Animal Experimentation of INIA (permit number 2013-015).

### Cells, viruses, infections and virus titrations

All virus manipulations were performed in our biosafety level 3 (BSL-3) facilities. HeLa cells [39] and 293T (ATCC CRL-3216) cells were grown in Dulbecco’s modified minimum essential medium (DMEM) supplemented with 10% fetal bovine serum, 2 mM glutamine and penicillin-streptomycin. The origin and passage history of the WNV lineage 1 strain NY99 (GenBank: KC407666.1) has been previously described [5], [40], [41]. WNV lineage 2 isolate SRB-Novi Sad/12 (GenBank: KC407673.1) corresponds to a virus isolated from a goshawk found dead in Serbia in 2012 [18]. The prototypic USUV strain SAAR-1776 (GenBank: AY453412.1) was used [42], [43], [44]. All viruses were amplified in Vero cells [41], [43]. Procedures for infections and virus titration in semisolid agarose medium have been described [41], [43].

### Antibodies and control sera

Mouse monoclonal IgG1 3.67G anti WNV E protein was from Millipore (Temecula, CA). Mouse monoclonal IgG1 to GFP (Roche, Manheim, Germany) was used as an irrelevant isotype-matched control antibody. Rabbit polyclonal serum against WNV M protein was from Imgenex (San Diego, CA). Control pooled sera from experimentally WNV-infected mice [45] or red-legged partridges [46], as well as appropriate negative controls from uninfected animals were used. In addition, the following secondary antibodies were employed: donkey anti-mouse IgG coupled to Alexa Fluor 594 (Invitrogen; Carlsbad, CA), goat anti mouse or anti-rabbit IgG coupled to horseradish peroxidase (Pierce Biotechnology; Rockford, IL), goat anti-bird IgG coupled to horseradish peroxidase (Bethyl Laboratories; Montgomery, TX), goat anti-mouse IgG coupled to 5 nm colloidal gold (British Biocell International; Cardiff, UK) and protein A coupled to 5 nm colloidal gold (Cell Microscopy Center; Department of Cell Biology, University Medical Center Utrech, The Netherlands).

### Molecular cloning

Plasmid pcDNA-WNV encoding the signal peptide from the C protein and the prM and E proteins from WNV-NY99 was constructed as follows. First, viral RNA was extracted from tissue culture medium of cells infected with WNV NY99 using the NucleoSpin Viral RNA Isolation kit (Macherey-Nagel GmbH & Co.; Düren, Germany). The cDNA encoding the last 25 amino acids from WNV anchored C protein (signal peptide) and complete prM and E glycoproteins was amplified by reverse transcription of viral RNA using SuperScript One-Step RT-PCR with Platinum Taq (Invitrogen) and oligonucleotide primers CAA*GCTAGC* GCCACC **ATG**AGCTCAAAACAAAAGAAAAGAGG and GTT*GGTACC* **CTA**AGCGTGCACGTT CACGG (restriction sites *Nhe*I and *Kpn*I, start and stop codons and a Kozac consensus sequence are indicated in italics, bold, and underlined, respectively). The resultant cDNA fragment was digested with *Nhe*I and *Kpn*I (New England Biolabs; Ipswich, MA) and ligated, using T4 DNA ligase (Promega; Madison, WI), with plasmid pcDNA 3.1 (+) (Invitrogen) digested with the same enzymes. The product of the ligation reaction was transformed into *E. coli* DH5α as described [47]. Positive colonies were obtained by selection in LB agar plates supplemented with ampicillin. Plasmid DNA was purified from bacteria through PureLink HiPure Plasmid Filter Maxiprep kit (Invitrogen). The nucleotide sequence of pcDNA-WNV was verified by automated nucleotide sequencing (Macrogen Europe; Amsterdam, The Netherlands).

### Transfections and stable cell line selection

HeLa cells were transfected with pcDNA or pcDNA-WNV using Lipofectamine PLUS Reagent (Invitrogen), as described by the manufacturer. For stable cell line selection, individual clones were obtained by limit dilution of HeLa cells transfected with pcDNA-WNV (48 h post-transfection) and grown in selective medium containing 500 µg/ml of G-418 (Gibco). Cell clones were screened for E protein expression by immunofluorescence and immunodot assay as described below.

### Purification of RSPs

Subviral particles were purified by sucrose gradient centrifugation as described [32], with minor variations. Briefly, culture medium clarified at 15000×g (30 min at 4°C) was centrifuged through a 20% sucrose cushion at 112000×g (3.5 h at 4°C). The pellet containing RSPs was resuspended in PBS and loaded onto a 12 ml 20–60% linear sucrose gradient (256000×g for 18 h at 4°C). Fractions of 0.5 ml were collected from the top of the gradient. For mouse experiments, peak fractions containing E protein were further purified by an additional sucrose gradient and peak fractions containing RSPs were pooled and dialyzed against PBS to remove the sucrose. The amount of proteins in purified RSP preparations was estimated by Bradford assay.

### Mouse experiments

Groups of 6–12 eight-week old *Swiss* female mice were inoculated intraperitoneally (i.p.) as described [36] with 200 µl of a PBS solution containing 0.2, 2 or 10 µg of RSPs adsorbed to 0.2% (vol/vol) of aluminium hydroxide, Al(OH)_3_ (Alhydrogel, InvivoGen, San Diego, CA). Control mice were inoculated with the same volume of PBS alone or PBS plus Al(OH)_3_. To analyze the induction of antibodies mice were bled at different time point post-immunization and post-infection (p.i.). Mice were challenged i.p. with 10^4^ PFU/mouse of each strain of WNV or USUV and monitored daily for signs of infection up to 15 days p.i. Animals were kept with *ad libitum* access to food and water and those exhibiting clear signs of disease as ruffled fur, hunching, hind limb weakness, and paralysis, were anesthetized and sacrificed, as were all surviving mice at the end of the experiment.

### Plaque reduction neutralization assays (PRNT)

PRNT were essentially performed on Vero cells as previously described [18]. Briefly, heat-inactivated pooled serum samples were diluted in culture medium and filtered through 0.22 µm. Neutralization was performed by incubating a fixed amount [100 plaque-forming unit (PFU)] of WNV NY99, SRB-Novi Sad/12 or USUV with two-fold serial dilutions (starting from 1∶20) of each serum for 1 h at 37°C. Then the mixture was adsorbed for 1 h to subconfluent Vero cell monolayers grown in six-well tissue culture plates. After adsorption, culture medium was removed and cells, overlaid with semisolid agarose medium, were incubated for 72 h, fixed, and stained with crystal violet [41], [43]. Titers were expressed as the reciprocal of the serum dilution that inhibited plaque formation by 90% (PRNT_90_), relative to samples incubated with negative control pooled sera.

### Enzyme-linked immunosorbent assays (ELISA)

Plates were coated with the antigen (RSPs or heat inactivated virus) diluted in coating buffer (0.015 M Na_2_CO_3_, 0.030 M NaHCO_3_; pH 9.6) and washed with PBS (RSPs) or PBS 0.05% Tween 20 (inactivated virus). For heat inactivation, culture medium of Vero cells infected with WNV-NY99 or USUV was inactivated at 65°C for 2 h and mixed with 30% PEG 8000 in 0.4 M NaCl at the ratio of 2∶1 (supernatant-PEG), overnight at 4°C with constant gentle rotation. Precipitate proteins were recovered by centrifugation at 8671×g (20 min at 4°C) dissolved in ELISA coating buffer, sonicated and stored at −20°C. After antigen coating, plates were blocked with 5% skimmed milk for 1****h at 37°C in PBS, washed and incubated with monoclonal antibodies or specific sera (1****h at 37°C) diluted in blocking solution, washed again and incubated with secondary antibodies coupled to horseradish peroxidase (1****h at 37°C). ELISA was developed using O-phenylene diamine dihydrochloride (Sigma; St. Louis, MO) and H_2_O_2_. Results were expressed as the absorbance at 492 nm (A492) or as positive/negative ratio (P/N) calculated by dividing the mean absorbance of the test serum by the absorbance of the negative control serum.

### Enzyme-linked immunodot assays

Cell culture medium was adsorbed to a nitrocellulose membrane by vacuum using a Bio-Dot apparatus (Bio-Rad; Hercules, CA). Membrane was blocked with 3% skimmed milk in PBS and incubated with primary antibodies diluted in 1% skimmed milk in PBS. After three washes with PBS the membrane was incubated with secondary antibodies coupled to horseradish peroxidase, washed and proteins were detected by chemiluminiscence using an ImageQuant LAS 4000 mini equipment (GE Healthcare, Buckinghamshire, United Kingdom).

### Western blot

Purified RSPs were mixed with Laemmli sample buffer, subjected to SDS-PAGE and electrotransferred onto a nitrocellulose membrane. Membrane was blocked with 3% skimmed milk in PBS, incubated with primary antibodies, washed three times with PBS 0.05% Tween 20, and subsequently incubated with secondary antibodies coupled to horseradish peroxidase. Membrane was washed three times and proteins were detected by chemiluminescence as described above.

### Immunofluorescence

HeLa cells grown on glass cover slips were transfected with pcDNA or pcDNA-WNV and 24 h post-transfection were fixed with 4% paraformaldehyde in PBS for 15 min. Cells were washed with PBS and permeabilized with BPTG (1% BSA, 0.1% Triton X-100, 1 M glycine in PBS) for 15 min, and then incubated with primary antibody diluted in 1% BSA in PBS. After washing, cells were incubated with fluorescently conjugated secondary antibody. Nuclei were stained with 4′,6-diamidino-2-phenylindole (DAPI) and samples were mounted with Fluoromount-G (SouthernBiotech, Birmingham, AL) and observed with an epifluorescencece microscope.

### Transmission electron microscopy

Negative staining of RSPs was performed as described for TBEV RSPs in dialyzed samples containing the peak of the gradient [32]. For immunolabelling and negative staining, samples were adsorbed to ionized collodion-carbon coated grids, washed with PBS and blocked with 10% fetal bovine serum in PBS for 5 min. Grids were incubated with primary antibodies diluted in 5% fetal bovine serum in PBS for 30 min, washed five times with PBS and incubated with anti-mouse IgG or protein A coupled to 5 nm colloidal gold diluted in 5% fetal bovine serum in PBS for 30 min. Samples were then fixed with 1% glutaraldehyde in PBS for 5 min, washed three times with bidistilled water and negatively stained with 1% uranyl acetate for 2 min. Samples were examined using a Jeol JEM-1010 electron microscope (Jeol, Japan) operated at 80 kV and images were acquired using a digital camera 4K64K TemCam-F416 (Tietz Video and Image Processing Systems GmbH, Gauting, Germany).

### Data analysis

To test the significance of the differences, analysis of the variance (ANOVA) was performed with statistical package SPSS 15 (SPSS Inc, Chicago IL) applying Bonferroni’s correction for multiple comparisons. Kaplan-Meier survival curves were analyzed by a logrank test using the statistical package GraphPad PRISM v.2.01 (GraphPad Software Inc.; La Jolla, CA). The median survival time (MST), defined as the time at which a survival curve crosses the 50% survival point, was calculated for every group of inoculated mice using GraphPad. Statistically significant differences were considered at P<0.05. One asterisk (*) or two asterisks (**) in the figures denote statistically significant differences with P<0.05 or P<0.005, respectively.

## Results

### Generation of a stable cell line expressing WNV envelope glycoproteins and characterization of the secreted antigens

**Figure 1:**
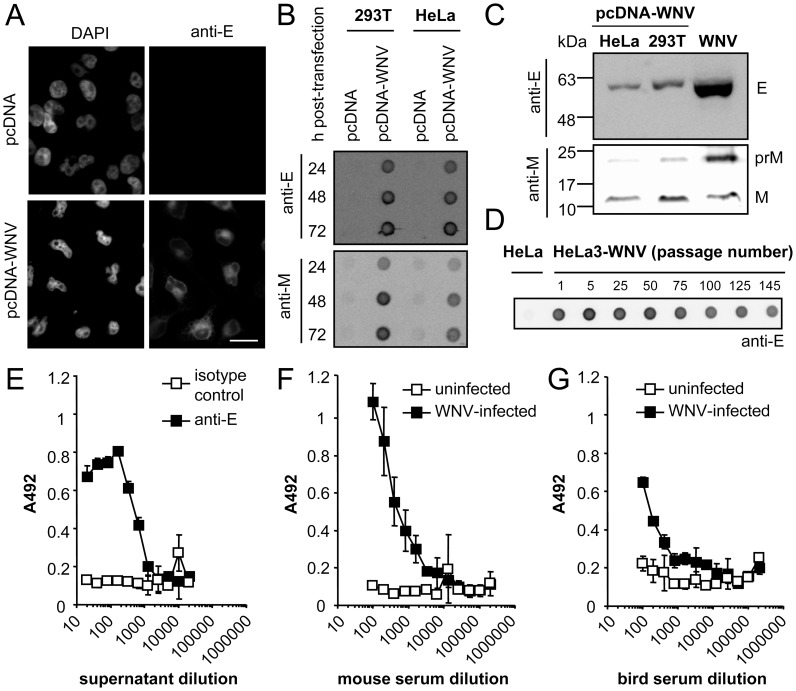
Expression of WNV proteins encoded by plasmid pcDNA-WNV. (A) Immunofluorescence analysis of E protein expression. HeLa cells were transfected with pcDNA or pcDNA-WNV and, 24 h later, fixed and processed for immunofluorescence using a monoclonal anti-E and a secondary antibody coupled to Alexa Fluor 594. Nuclei were stained with DAPI. Scale bar 20 µm. (B) Analysis of WNV protein expression in the culture medium from 293T or HeLa cells transfected with pcDNA-WNV by enzyme-linked immunodot assay. Both cell lines were transfected with pcDNA or pcDNA-WNV and, 24, 48 or 72 h later, the culture medium was adsorbed onto a nitrocellulose membrane and detected using a monoclonal anti-E or a polyclonal anti-M and the corresponding secondary antibodies coupled to horseradish peroxidase. (C) Western blot analysis of WNV proteins released to the culture medium after transfection with pcDNA-WNV. HeLa or 293T cells were transfected with plasmid pcDNA-WNV and the viral proteins in the culture medium were purified by ultracentrifugation through a 20% sucrose cushion. Proteins were blotted with the antibodies used in panel (B). A control lane containing purified WNV from WNV-infected Vero cells was included. (D) Expression of E protein in the culture medium of HeLa cells stably transfected with pcDNA-WNV (HeLa3-WNV). The E protein content in the culture medium from different passages of HeLa3-WNV cells was determined by an enzyme-linked immunodot assay using a monoclonal antibody against E protein. (E) Analysis by ELISA of the reactivity of WNV antigens released to culture medium of HeLa3-WNV cells. ELISA plates were coated with two-fold serial dilutions of culture medium from HeLa3-WNV cells and incubated either with a monoclonal anti-E or an irrelevant isotype-matched control antibody. Samples were incubated with appropriate secondary antibodies and developed using O-phenylene diamine dihydrochloride. ELISA results were expressed as the absorbance at 492 nm (A492). (F, G) Analysis of the reactivity of viral proteins secreted by HeLa3-WNV with pooled sera from experimentally infected mice (F) or birds (G). ELISA plates were coated with a fixed dilution of HeLa3-WNV culture medium and incubated with two-fold serial dilutions of sera from WNV-infected mice or red-legged partridges and the ELISA was performed as described in (E). Pooled sera from control uninfected animals were included as negative controls.

Plasmid pcDNA-WNV encoding the 25 last amino acids of the C-terminal hydrophobic sequence of the WNV-NY99 C protein followed by the complete sequence of the prM and E proteins was constructed (see Material and Methods for details). The expression of WNV antigens following transfection of pcDNA-WNV was verified by immunofluorescence staining using a monoclonal antibody directed to the E glycoprotein (Figure 1A). In order to analyze the secretion of WNV antigens to the extracellular medium, the supernatant from HeLa or 293T cells transfected with empty vector (pcDNA) or with pcDNA-WNV was harvested at different times post-transfection and the presence of viral antigens was determined by an enzyme-linked immunodot assay (Figure 1B). Positive signals were obtained with the anti-E and the anti-M antibodies at 24, 48 and 74 h post-transfection of both cell lines with pcDNA-WNV but not with the empty vector. The intensity of the signal increased with time pointing to an accumulation of viral antigens in the medium of transfected cultures.

Since processing of flavivirus structural protein precursors is mediated by cellular proteases [48], the correct cleavage of the E and M proteins was investigated. To this end, culture medium from transfected cultures was concentrated by ultracentrifugation through a 20% sucrose cushion and analyzed by Western blot (Figure 1C). In these experiments, infectious WNV produced in Vero cells and concentrated through a sucrose cushion was included as a marker for the molecular size of WNV proteins. Using an anti-E antibody, a single band with a mobility expected for the E glycoprotein was detected in samples from culture medium of both 293T and HeLa cells transfected with pcDNA-WNV. When an anti-M antibody was used, the mature M protein was detected in samples from HeLa and 293T cells, and an additional band, similar to that of WNV particles, corresponding to the prM protein was also observed. As transfection with pcDNA-WNV resulted in correct expression and processing of WNV antigens, a HeLa cell clone (HeLa3-WNV) stably transfected with this plasmid was obtained by limiting dilution and selection with G-418. HeLa3-WNV cell line retained the expression of WNV proteins upon freeze and thawing and over serial passages. In fact, the expression of WNV E protein in the culture medium could be detected up to, at least, 145 passages (Figure 1D). When culture medium from HeLa3-WNV cultures was further evaluated by ELISA using a monoclonal antibody to the E protein, a positive signal could be detected up to a 1∶640 dilution (Figure 1E). The viral antigens contained in the culture medium of HeLa3-WNV were also recognized by pools of sera from WNV-experimentally infected mice and birds (red-legged partridges) in ELISA (Figures 1F and G). These results confirm that HeLa3-WNV cells released to the culture medium viral proteins recognized by antibodies from infected-animals of different species.

**Figure 2:**
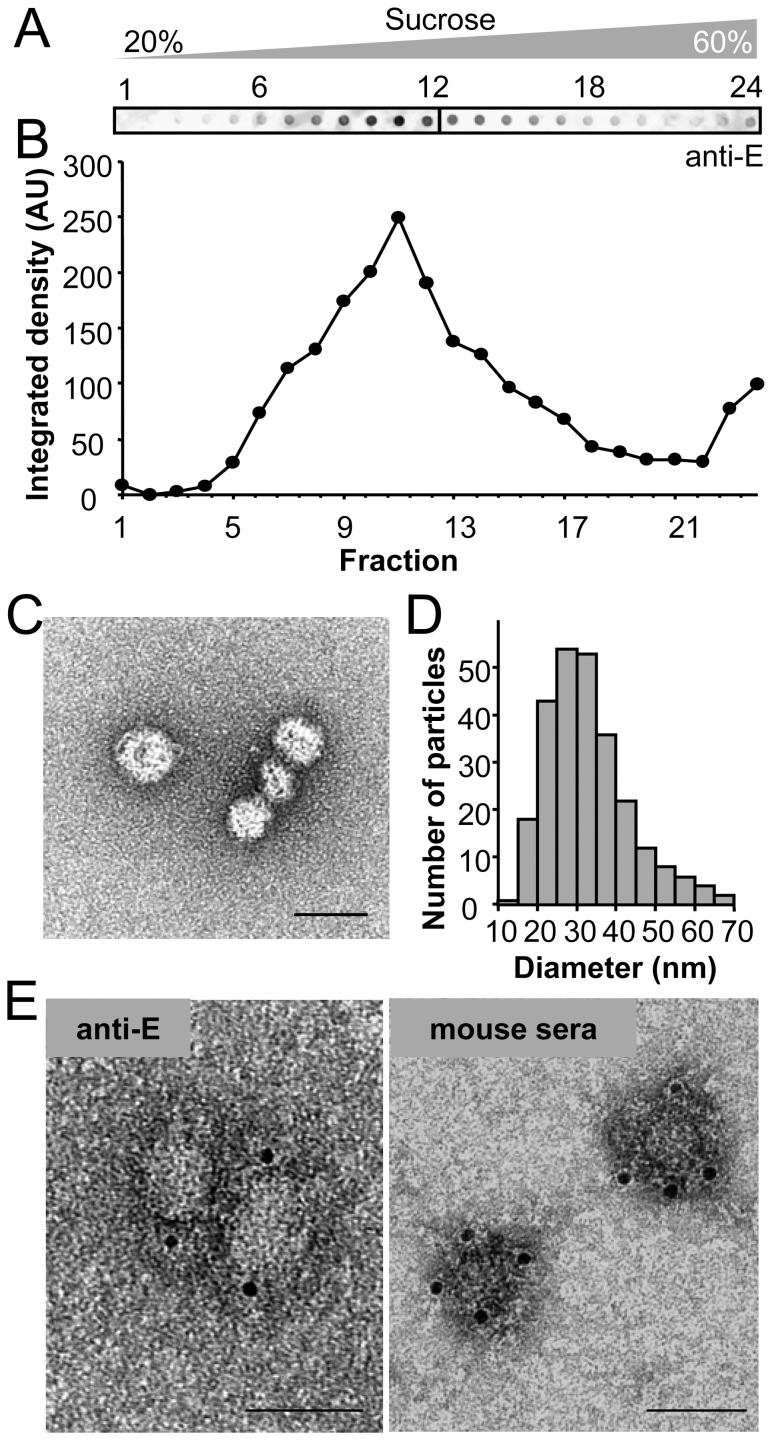
Structural analyses of WNV antigens secreted by HeLa3-WNV cells. (A) WNV proteins released to the culture medium of HeLa3-WNV cells were purified by centrifugation through a 20% sucrose cushion and loaded onto a 20–60% linear sucrose gradient. The amount of E glycoprotein in each fraction of the gradient was determined by enzyme-linked immunodot assay using a monoclonal antibody against the WNV E protein. (B) Quantification of the integrated density in each dot corresponding to the gradient fractions shown in panel (A). (C) Negative stained TEM image of purified WNV RSPs contained in peak fraction 11 of the gradient in panel (A). Scale bar: 50 nm. (D) Histogram showing the diameter of a total of 259 RSPs visualized by transmission electron microscopy and negative staining as in panel (C). (E) Purified RSPs were immunolabelled with either a monoclonal anti-E (left panel) or a pooled sera from WNV-infected mice (right panel), incubated with protein A or anti-mouse IgG coupled to 5 nm diameter colloidal gold. Samples were negatively stained and observed by transmission electron microscopy. Scale bars: 50 nm.

To verify that the secreted proteins produced by HeLa3-WNV were arranged in RSPs, culture medium was concentrated through a 20% sucrose cushion followed by equilibrium centrifugation into a linear 20–60% sucrose gradient (Figure 2A and B). The analysis of the gradient fractions by immunodot revealed a single peak of E protein that contained smooth spherical particles, as observed by negative staining and transmission electron microscopy (Figure 2C). The size of these particles varied, the most abundant particles ranging 20–40 nm in diameter, being the mean diameter of 33±10 nm (Figure 2D). The presence of WNV proteins in these structures was further confirmed by negative staining and immunolabelling of purified RSPs incubated with a monoclonal anti-E antibody (Figure 2E, left panel). In addition, these structures were also recognized by pooled sera from mice experimentally infected with WNV (Figure 2E, right panel). This further confirmed that RSPs displayed viral epitopes that could be recognized by the antibodies developed in WNV-infected animals. Taken together these results show that HeLa3-WNV cells stably transfected with pcDNA-WNV produce WNV RSPs displaying viral proteins.

### Immunogenicity of the secreted WNV RSPs

**Figure 3:**
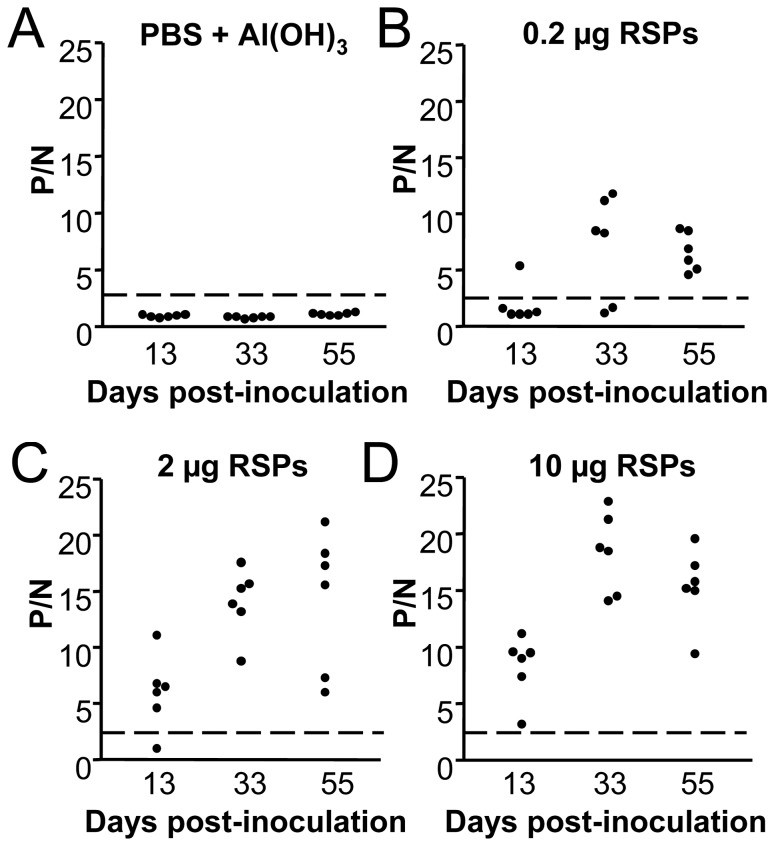
Induction of anti-WNV IgG in mice immunized with WNV-RSPs. Groups of six *Swiss* mice were sham immunized by inoculation with PBS plus Al(OH)_3_ (A) or immunized with 0.2 (B), 2 (C) or 10 (D) µg per mouse of purified RSPs formulated with Al(OH)_3_. Mice were boosted twice with a dose of 0.2 µg (animals initially inoculated with 0.2 µg) or 2 µg (animals initially inoculated with 2 or 10 µg) of RSPs at 19 (1^st^ boost) and 41 (2^nd^ boost) days post-immunization. Mice were bled about two weeks after each immunization (13, 33 and 55 days post-inoculation) and the presence of anti-WNV IgGs was assayed by an indirect ELISA using plates coated with heat inactivated WNV. ELISA titers are expressed as P/N ratios (see Material and methods). Each point of the graph represents the P/N for a single animal. Dashed lines denote the threshold of ELISA assays (P/N = 2.5).

**Figure 4:**
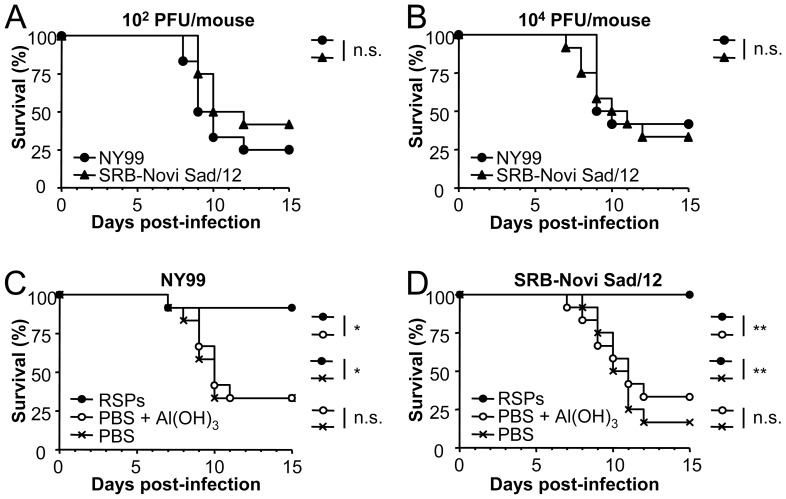
Protection conferred by RSPs against WNV strains from lineage 1 or 2. (A, B) Comparison of the virulence in mice of two distinct WNV isolates. Groups of 12 eight-week old *Swiss* female mice were infected in parallel with 10^2^ (A) or 10^4^ (B) PFU/mouse of WNV NY99 (lineage 1) or SRB-Novi Sad/12 (lineage 2) and the mortality was recorded along time. (C, D) Protection conferred by the immunization with RSPs against a lethal WNV challenge. Groups of 12 mice were inoculated intraperitoneally with PBS alone, PBS plus Al(OH)_3_, or 2 µg of purified RSPs plus Al(OH)_3_ and challenged (two weeks post-inoculation) with 10^4 ^PFU/mouse of WNV NY99 (C) or SRB-Novi Sad/12 (D). Statistically significant differences are indicated by one asterisk (*) for P<0.05 or two asterisks (**) for P<0.005. n.s. denotes not statistically significant differences.

Groups of six *Swiss* mice were inoculated intraperitoneally with 0.2, 2 or 10 µg per mouse of purified RSPs formulated with Al(OH)_3_ as adjuvant, and boosted twice with a dose of 0.2 µg (animals initially inoculated with 0.2 µg) or 2 µg (animals initially inoculated with 2 or 10 µg) of RSPs at 19 (1^st^ boost) and 41 (2^nd^ boost) days post-inoculation. A group of control animals inoculated with PBS plus Al(OH)_3_ was included. Mice were bled at 13, 33, and 55 days post-immunization. The presence of anti-WNV IgGs was assayed by an indirect ELISA using plates coated with heat inactivated WNV. Control animals receiving PBS plus Al(OH)_3 _did not show detectable levels of anti-WNV IgG in serum samples throughout the experiment (Figure 3A). Conversely, one of the six animals receiving 0.2 µg of RSPs was positive after the first inoculation (hereinafter referred to as inoculation) (Figure 3B) and 4 showed positive results after the first boost, while all the animals developed anti WNV-IgG two weeks after the second boost. The 2 µg dose was enough to induce anti-WNV IgG in 5 out of 6 (83%) animals after inoculation, being all positive after the first boost and showing a trend for titer increase upon the second boost (Figure 3C). All mice immunized with 10 µg of RSPs developed anti-WNV IgG and their titers increased after boosting (Figure 3D). The induction of WNV-neutralizing antibodies was analyzed by PRNT (Table 1). Control mice receiving PBS plus adjuvant did not show WNV-neutralizing antibodies, whereas mice immunized with 0.2 µg of RSPs displayed detectable levels of neutralizing antibodies after the first boost. Both groups of animals receiving 2 or 10 µg of RSPs exhibited low although detectable neutralizing antibody levels after inoculation (1∶20), which were markedly increased after first boost up to values >1∶640. These results confirmed that RSPs produced by the HeLa3-WNV cell line could work as suitable immunogens since they were able to induce anti-WNV antibodies after a single administration. As no marked differences in the induction of specific IgG and neutralizing antibodies were observed between animals immunized with 2 or 10 µg of RSPs, a 2 µg dose was selected for subsequent experiments involving viral challenge.

**Table 1:** Induction of WNV-neutralizing antibodies in mice immunized with different doses of RSPs.

	Neutralizing antibody titer (PRNT_90_) on days post-inoculation^a^		
	13	33	55
PBS+ Al(OH)_3_	Neg	Neg	Neg
RSPs 0.2 µg	Neg	1∶230	1∶256
RSPs 2 µg	1∶20	>1∶640	>1∶640
RSPs 10 µg	1∶20	>1∶640	>1∶640

a Serum neutralization titers of pooled sera from six mice in each group correspond to PRNT_90_ values for WNV NY99. Samples were taken 13 days post-inoculation, two weeks after first boost (33 days post-inoculation) and two weeks after second boost (55 days post-inoculation). Neg, negative (<1∶20).

### Evaluation of protection conferred by a single dose of RSPs against infection with two genetically divergent strains of WNV

WNV-induced death of experimentally inoculated mice is associated with the virulence of the viral strain used [49], [50], [51]. Along this line, we first assessed the virulence in mice of the lineage 2 strain SRB-Novi Sad/12. This viral strain was recently isolated from a dead bird in Serbia during the outbreak of WNV neuroinvasive disease in East Europe in 2012 [18], [52], and was likely responsible of the first neuroinvasive human cases described in the region. In these experiments the highly neurovirulent lineage 1 strain NY99 often used as a challenge strain in vaccine testing was included for comparison. Mice were inoculated intraperitoneally with 10^2^ or 10^4^ PFU of WNV NY99 or SRB-Novi Sad/12. Both groups of mice exhibited signs of WNV disease as ruffled fur or hunching and no significant differences related to the mortality induced by both strains of WNV were noticed (Figure 4A and B), pointing to SRB-Novi Sad/12 as a suitable highly virulent challenge strain of WNV lineage 2.

The potential of RSPs as vaccine candidates was then evaluated by inoculating mice with a single dose of 2 µg of RSPs combined with Al(OH)_3_, which was the lowest dose tested that induced detectable levels of neutralizing antibodies after a single inoculation (Table 1). Animals were challenged with 10^4^ PFU of WNV NY99 or SRB-Novi Sad/12 two weeks post-inoculation (Figures 4C and D). The percentage of surviving mice inoculated with RSPs and challenged with WNV NY99 (Figure 4C) was 91.6%, a value significantly higher than that of mice inoculated with PBS or PBS plus Al(OH)_3_ (33.3%), as was the MST that also increased (>15 days for RSPs, 10 days for PBS and PBS plus Al(OH)_3_). Interestingly, all mice (100%, MST >15 days) inoculated with RSPs survived the challenge with the heterologous WNV SRB-Novi Sad/12, whereas only 33.3% (MST of 11 days) and 16.7% (MST of 10.5 days) of mice inoculated with PBS plus Al(OH)_3 _or PBS alone survived this challenge, respectively (Figure 4D).

Due to the relevance of neutralizing antibodies in WNV protection, the induction of such antibodies was determined in the mice shown in Figure 4. Animals immunized with RSPs displayed at 3 and 4 days post-challenge neutralizing antibodies titers higher than those of control mice inoculated with PBS or PBS plus Al(OH)_3,_ regardless the WNV strain used for challenge (Table 2). At day 15 post-challenge the neutralizing antibody titers were overall higher than 1∶640 for all groups of surviving mice, including both control and immunized animals, except for neutralization of NY99 by mice inoculated with PBS and challenged with NY99.

**Table 2:** Induction of WNV-neutralizing antibodies in mice inoculated with RSPs upon challenge with WNV.

		Neutralizing antibody titer (PRNT_90_) on days post-challenge^b^		
Challenge strain	Group^a^	3	4	15
NY99	PBS	Neg	1∶26	1∶302
	PBS+Al(OH)_3_	Neg	1∶46	>1∶640
	RSPs	1∶120	1∶149	>1∶640
SRB-Novi Sad/12	PBS	Neg	1∶68	>1∶640
	PBS+Al(OH)_3_	Neg	1∶25	>1∶640
	RSPs	1∶99	1∶165	>1∶640

a Animals were immunized with a single dose containing 2 µg of RSPs and challenged two weeks post-inoculation.

b Serum neutralization titers of pooled sera from twelve mice in each group. Neutralization against the homologous viral strain selected for challenge (NY99 or SRB-Novi Sad/12) are displayed. Neg, negative (<1∶20).

### Analysis of the cross-reactive humoral response against USUV in mice immunized with WNV RSPs

**Figure 5:**
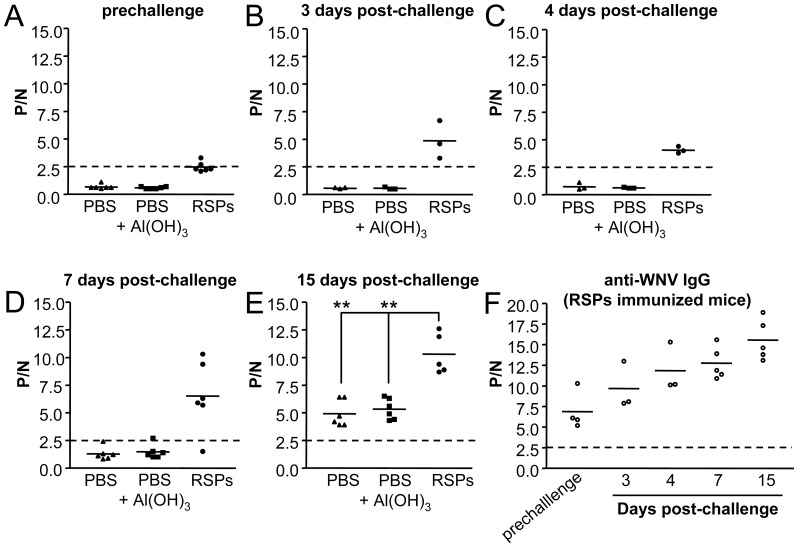
Induction of cross-reactive anti-USUV or anti-WNV IgGs in mice immunized with WNV RSPs and challenged with USUV. Groups of six eight-weeks old *Swiss* female mice were sham immunized intraperitoneally with PBS or PBS plus Al(OH)_3_ or immunized with 2 µg of WNV RSPs plus Al(OH)_3_ and challenged with 10^4^ PFU of USUV two weeks later. (A to E). Anti-USUV antibodies in blood samples, collected at the days post-challenge indicated in each case, detected by an indirect ELISA using plates coated with heat inactivated USUV. (F) Anti-WNV antibodies in serum collected at different days post-challenge were detected by an indirect ELISA using plates coated with heat inactivated WNV. ELISA titers are expressed as P/N ratios. Each point of the graph represents the P/N for a single animal. Solid lines represent the mean P/N of each group. Dashed lines denote the threshold of ELISA assays (P/N = 2.5). Statistically significant differences are indicated by two asterisks (**) for P<0.005.

Since immunization with WNV-RSPs induced a protective humoral response against genetically distant viral strains of WNV such as SRB-Novi Sad/12, and different flaviviruses share common antigenic determinants, the potential cross-reaction of this immunization against other related flaviviruses was evaluated using USUV. First, the induction of cross-neutralizing antibodies after inoculation with WNV-RSPs was determined. While no anti-USUV neutralizing antibodies were detected in PBS plus Al(OH)_3_ sham immunized mice, or in mice immunized with 0.2 µg of WNV-RSPs, detectable titers were found in animals immunized with 2 or 10 µg of WNV-RSPs after two boosts (Table 3). Furthermore, sera from mice immunized with a single dose of 2 µg of WNV-RSPs and challenged with WNV NY99 or SRB-Novi Sad/12 showed titers of neutralizing antibodies at 15 days p.i. higher than those of non-immunized animals (PBS alone or PBS plus Al(OH)_3_; Table 4). To address whether the infection with USUV could mimic some features of a challenge with WNV and stimulate the production of cross-reactive antibodies in animals immunized with WNV-RSPs, groups of six eight-week old *Swiss* mice were inoculated with PBS, PBS plus Al(OH)_3_ or 2 µg of WNV-RSP and subsequently challenged with 10^4^ PFU of USUV per mouse at 15 days post-immunization. This USUV challenge did not induce mortality in adult mice in any of the groups tested. When the outcome of the humoral response against USUV was evaluated by ELISA (Figure 5) it was observed that mice immunized with WNV-RSPs displayed low although detectable levels of cross-reactive IgG with USUV (mean P/N of 2.5±0.4) in contrast to mice inoculated with PBS or PBS plus adjuvant (mean P/N of 0.7±0.2 and 0.6±0.1, respectively) before challenge with USUV (Figure 5A). The level of IgGs increased after challenge with USUV (days 3, 4 and 7 post-challenge). in WNV-RSPs inoculated animals, whereas only one animal of each control group (PBS or PBS plus Al(OH)_3_) displayed very low, but detectable levels of anti-USUV IgG at 7 days p.i. (Figure 5 B, C and D), confirming that induction of IgG against USUV was primed by immunization with WNV-RSPs. At the end of the experiment, 15 days p.i. (Figure 5E), all USUV challenged mice showed detectable levels of anti-USUV IgG, although these levels were significantly higher for mice previously inoculated with WNV-RSPs (mean P/N of 10.3±1.8) than for those inoculated with PBS (mean P/N of 4.9±1.2) or PBS plus Al(OH)_3_ (mean P/N of 5.3±0.9). The presence of anti-WNV antibodies was also confirmed in these mice immunized with WNV-RSPs and challenged with USUV (Figure 5F). The levels of anti-WNV IgG increased with time following USUV infection, further supporting that challenge with USUV acted as a boost for the humoral response induced by inoculation with WNV-RSPs. Likewise, mice immunized with WNV-RSPs developed higher USUV neutralizing antibody titers upon challenge (day 15) than those of control animals (Table 5). As expected, these animals exhibited a higher increase on anti-WNV NY99 or SRB-Novi Sad/12 neutralizing antibody titers, supporting that infection with USUV boosted the antibody response elicited after immunization with WNV-RSPs.

**Table 3:** Induction of crossreactive USUV-neutralizing antibodies in mice immunized with different doses of WNV RSPs.

	Neutralizing antibody titer (PRNT_90_) on days post-inoculation^a^		
	13	33	55
PBS+Al(OH)_3_	Neg	Neg	Neg
RSPs 0.2 µg	Neg	Neg	Neg
RSPs 2 µg	Neg	Neg	1∶169
RSPs 10 µg	Neg	Neg	1∶165

a Serum neutralization titers of pooled sera from six mice in each. Samples were taken 13 days post-inoculation, two weeks after first boost with 2 µg of RSPs (33 days post-inoculation) and two weeks after second boost (55 days post-inoculation). Neg, negative (<1∶20).

**Table 4:** Induction of cross-neutralizing antibodies against USUV in mice inoculated with WNV RSPs upon challenge with WNV.

		Neutralizing antibody titer (PRNT_90_) against USUV^b^			
Challenge strain	Group^a^	Pre-challenge	3	4	15
NY99	PBS	Neg	Neg	1∶75	1∶86
	PBS+Al(OH)_3_	Neg	Neg	Neg	Neg
	RSPs	Neg	Neg	1∶75	>1∶640
SRB-Novi Sad/12	PBS	Neg	Neg	Neg	1∶44
	PBS+Al(OH)_3_	Neg	Neg	Neg	1∶20
	RSPs	Neg	Neg	Neg	1∶116

a Animals were immunized with a single dose containing 2 µg of RSPs and challenged two weeks post-inoculation.

b Serum neutralization titers of pooled sera from six mice in each group correspond to PRNT_90_ values. Neg, negative (<1∶20).

**Table 5:** Induction of neutralizing antibodies in mice immunized with WNV RSPs and infected with USUV.

	Neutralizing antibody titer (PRNT_90_) against USUV or WNV strains on days post-challenge^a^			
	4		15	
	USUV	WNV NY99/SRB-Novi Sad/12	USUV	WNV NY99/SRB-Novi Sad/12
PBS+ Al(OH)_3_	Neg	Neg/Neg	Neg	Neg/Neg
RSPs^b^	Neg	1∶90/1∶58	1∶45	1∶228/>1∶640

a Serum neutralization titers against the viruses indicated of pooled sera from 3 and 6 mice on day 4 and 15 post-challenge, respectively. Neg, negative (<1∶20).

b Animals were immunized with a single dose containing 2 µg of WNV RSPs and challenged with USUV at two weeks post-inoculation.

## Discussion

Co-expression of flavivirus glycoproteins in transfected cells induces production of RSPs that are secreted into the culture medium [32], [33], [34], [35], [53]. A widely used strategy to achieve this goal is to express the tandem prM-E protein preceded by a signal peptide as the C-terminal hydrophobic sequence of the C protein of the same or another flavivirus [54], [55] or by a different signal peptide, even from an unrelated virus [34]. Accordingly, plasmid pcDNA-WNV encoding the WNV C signal peptide followed by the prM-E was constructed and shown to correctly express the processed proteins. In order to provide noninfectious sources of flaviviral antigens, a variety of cell lines continuously expressing different prM and E glycoproteins have been reported [33], [34], [37], [54]. In this study we have generated a HeLa cell line stably transfected with plasmid pcDNA-WNV as a noninfectious and easy to use platform for the production of RSPs. The physical properties of these particles as well as their antigenicity were characterized by means of ELISA, western blot, ultracentrifugation and transmission electron microscopy. It has been reported that minor different subpopulations of flaviviral RSPs can be separated by varying the purification conditions and that they could differ in maturation status and immunogenicity [33], [56]. Our results show that using equilibrium sucrose density gradient centrifugation a peak of protein containing RSPs that vary in size but have a mean diameter of 30 nm was observed, a feature consistent with previous findings derived from the structural characterization of flavivirus RSPs [32], [35], [53]. The antigenicity of these structures was confirmed by ELISA and immunogold electron microscopy using anti-E antibodies or sera from WNV-infected mice and birds.

A previous report had shown that inoculation of RSPs could protect mice against challenge with a homologous lineage 1 WNV strain after two administrations [33]. However, single dose vaccines are desirable, especially for application during outbreaks, in vaccination campaigns in poorly industrialized countries or for wild animals. In addition to this, the recent epidemiologic scenario of WNV has highlighted the need for verifying the efficacy of available vaccines against viruses from lineages 1 and 2 [57], [58], [59]. Having in mind these considerations, the protection conferred by immunization with our WNV RSPs against lethal challenge with WNV virulent strains from both lineages was investigated. Searching for an appropriate single dose vaccine formulation, the immunogenicity of RSPs produced by the stable cell line was assayed using an adjuvant, Al(OH)_3_, widely used for animal and human vaccine formulation to enhance humoral immune response [60], [61], that constitutes a key player in WNV protection [62], [63]. A single inoculation of RSP-based vaccine was enough to induce circulating anti-WNV IgG and detectable titers of neutralizing antibodies, which are known to be crucial to confer protection against WNV [62], [63]. Mice inoculated with a single dose of the RSP-based vaccine showed a very high protection rate (91.6 and 100%, respectively) against lethal challenge with homologous and heterologous WNV strains. This protection was associated with the detection upon challenge of higher neutralizing antibody titers in mice immunized with RSPs than those observed in sham immunized animals; indicating that protection induced by the RSPs was based on the induction of neutralizing antibodies. These results evidence the feasibility of the RSP-based formulation here described as vaccine candidates capable to induce protection against WNV lineages 1 and 2. This finding is in agreement with the cross-protection against lineages 1 and 2 conferred by different WNV vaccines [57], [58], [59], [64].

Vaccines against prominent flaviviruses such as YFV, JEV, TBEV, WNV or DENV are currently available or under development. Conversely, the development of vaccines specific for flaviviruses that, so far, cause less-frequent diseases, such as USUV, is unlikely [65]. In this regard, the cross-reactivity of WNV vaccines against USUV could be helpful for the control of USUV and other emerging pathogens. In fact, the potential of vaccines against flaviviruses of the JEV serocomplex to induce cross-reactive protective responses against antigenically related viruses has been recently reviewed [31]. Since WNV and USUV share multiple ecologic features, such as hosts and vectors, and co-circulate in both Africa and Europe (see Introduction), the potential induction of cross-reactive antibodies following vaccination with WNV-RSPs was analyzed. Vaccination of adult mice with WNV-RSPs induced low, although detectable, levels of circulating IgG cross-reactive with USUV, and a boost on these levels was observed upon infection with USUV. The cross-reactivity of these antibodies with WNV was confirmed in ELISA and neutralization tests. Unfortunately, protection studies of mice immunized with WNV-RSPs against USUV challenge are impaired as no lethal challenge model for USUV is available. The levels of cross-reactive antibodies found were low and, therefore, further work is required to elucidate if RSP-based vaccines can effectively protect against antigenically related lethal flaviviruses. Nevertheless, it should be noted that vaccines inducing low or undetectable levels of neutralizing antibodies can protect against flavivirus infection thanks to the anamnestic neutralizing antibody response [66]. In addition, the induction of low levels of circulating neutralizing antibody titers (1∶10) against different flaviviruses constitutes a widely used correlate with protection among vaccinated humans (reviewed in reference [67]). On the other hand, although cross-reactivity could be disadvantageous in vaccine approaches against some flavivirus from other groups due to antibody dependent enhancement of infection effect, this phenomenon has not been documented among viruses from the JEV serocomplex (in which WNV and USUV are included). Therefore, the possibility of a cross-reactive vaccine for flaviviruses within this group should be seriously considered, even though not all flavivirus vaccines against JEV serocomplex viruses tested induce effective cross-reacting responses [31]. For instance, there are contradictory evidences supporting or not that JEV vaccines can protect against infection with WNV or MVEV [65], [68], [69], pointing to the necessity to evaluate each specific situation.

In this report, the feasibility of WNV-RSPs as a safe, noninfectious, second-generation vaccine candidate against WNV has been documented. The results here presented show that a single dose of WNV-RSPs can induce a protective immune response against WNV strains from lineage 1 and 2. In addition, WNV-RSPs act as immunogens that can induce low, although detectable, cross-reactive humoral responses against related flaviviruses as USUV.
